# Diastolic Blood Pressure J-Curve Phenomenon in a Tertiary-Care Hypertension Clinic

**DOI:** 10.1161/HYPERTENSIONAHA.119.12787

**Published:** 2019-08-19

**Authors:** Stefanie Lip, Li En Tan, Panniyammakal Jeemon, Linsay McCallum, Anna F. Dominiczak, Sandosh Padmanabhan

**Affiliations:** 1 From the Institute of Cardiovascular and Medical Sciences, University of Glasgow, United Kingdom (S.L., L.E.T., P.J., L.M., A.F.D., S.P.); 2 Sree Chitra Tirunal Institute for Medical Sciences and Technology, Trivandrum, Kerala, India (P.J.).

**Keywords:** blood pressure, heart failure, humans, hypertension, myocardial ischemia

## Abstract

Concerns exist regarding the potential increased cardiovascular risk from lowering diastolic blood pressure (DBP) in hypertensive patients. We analyzed 30-year follow-up data of 10 355 hypertensive patients attending the Glasgow Blood Pressure Clinic. The association between blood pressure during the first 5 years of treatment and cause-specific hospital admissions or mortality was analyzed using multivariable adjusted Cox proportional hazard models. The primary outcome was a composite of cardiovascular admissions and deaths. DBP showed a U-shaped association (nadir, 92 mm Hg) for the primary cardiovascular outcome hazard and a reverse J-shaped association with all-cause mortality (nadir, 86 mm Hg) and noncardiovascular mortality (nadir, 92 mm Hg). The hazard ratio for the primary cardiovascular outcome after adjustment for systolic blood pressure was 1.38 (95% CI, 1.18–1.62) for DBP <80 compared with DBP of 80 to 89.9 mm Hg (referrant), and the subdistribution hazard ratio after accounting for competing risk was 1.33 (1.17–1.51) compared with DBP ≥80 mm Hg. Cause-specific nonfatal outcome analyses showed a reverse J-shaped relationship for myocardial infarction, ischemic heart disease, and heart failure admissions but a U-shaped relationship for stroke admissions. Age-stratified analyses showed DBP had no independent effect on stroke admissions among the older patient subgroup (≥60 years of age), but the younger subgroup showed a clear U-shaped relationship. Intensive blood pressure reduction may lead to unintended consequences of higher healthcare utilization because of increased cardiovascular morbidity, and this merits future prospective studies. Low on-treatment DBP is associated with increased risk of noncardiovascular mortality, the reasons for which are unclear.

## Abstract 2

Supplemental Digital Content is available in the text.

Hypertension is the single largest modifiable risk factor for cardiovascular events,^1^ with treatment guidelines informed by unequivocal evidence for benefit with pharmacological blood pressure (BP) lowering in randomized controlled trials.^2^ However, one of the unresolved controversies in hypertension management over the last 40 years is the issue of the diastolic J curve^3,4^—reduction in diastolic blood pressure (DBP) <85 to 90 mm Hg was not associated with lower risks, and reduction <70 to 80 mm Hg resulted in increased coronary events but not stroke.^5,6^ Most of the evidence for the J curve comes from observational studies or post hoc analyses of randomized controlled trials.^7–9^ The studies providing evidence for the J curve were also plagued by reverse causality and regression dilution bias.^10,11^ Moreover, the natural decrease in DBP with age after 55 years of age causes a late-life bias and a nonlinear distortion between mortality risk and risk factor in mixed-age adult follow-up cohorts.^12,13^

In the Framingham and the CALIBER (Cardiovascular Disease Research Using Linked Bespoke Studies and Electronic Health Records) studies, the diastolic J curve was observed only in the presence of high systolic blood pressure (SBP; ie, high pulse pressure).^14–16^ However, in the CLARIFY registry (Prospective Observational Longitudinal Registry of Patients with Stable Coronary Artery Disease) registry, the diastolic J curve persisted in patients with coronary heart disease, even among those in the lowest pulse pressure range.^17^ Additionally, a recent post hoc analysis of high-risk patients from the ONTARGET (Ongoing Telmisartan Alone and in Combination with Ramipril Global Endpoint) and TRANSCEND (Telmisartan Randomised Assessment Study in Angiotensin Converting Enzyme Inhibitor Intolerant Subjects with Cardiovascular Disease) trials showed that both achieved SBP <120 mm Hg and DBP <70 mm Hg were associated with increased cardiovascular and all-cause deaths,^18^ even when the achieved SBP was within optimal range of 120 to 140 mm Hg.^19^

The clinical importance of the role of diastolic J curve, if present, continues to be a matter of debate primarily because of the conflicting reports noted above and focus on intensive BP lowering in recent guidelines.^20,21^ The 2018 European Society of Cardiology/European Society of Hypertension guidelines recommend a DBP target of <80 mm Hg in only those without established coronary heart disease.^21^ Currently, <50% of patients treated for hypertension achieve a target office SBP <140 mm Hg; however, with the new guidelines emphasizing a greater focus on lower target BP, more clarity is required on the risks of the diastolic J curve, if present.

The key objective of the present study was to study the associations of on-treatment office DBP measured during a 5-year period on cause-specific cardiovascular hospital admissions and mortality during a 30-year follow-up period in a real-life hypertensive cohort.

## Materials and Methods

Data will be available on request to third parties through the Glasgow Safe Haven (details available at https://www.nhsggc.org.uk/about-us/professional-support-sites/nhsggc-safe-haven/).

### Study Setting and Study Population

The Glasgow Blood Pressure Clinic (GBPC) located in the Greater Glasgow area is the largest and the main specialist hypertension clinic in Glasgow, which provides secondary- and tertiary-level service to patients with hypertension. Further details on the study population and measurements have been described previously.^22^ In brief, patients were referred to the GBPC if their BPs were not controlled in primary care with at least 3 drugs or if there was evidence of high-risk factors such as early-onset hypertension and a family history of premature CVD. Structured instruments were used to collect data from all patients attending the clinic and were stored electronically in a single computerized database, which contains information on 16 011 patients attending the clinic from 1969 to 2011. The West of Scotland Research Ethics Service of the National Health Service (NHS) has approved analysis of anonymized data from the GBPC database (11/WS/0083). The ethics committee approves analysis of anonymized clinical data of patients collected during routine clinical care for patient care and for patient benefit. The data are held securely in an NHS-approved secure site, and anonymized extracts are permitted for analyses. All patients attending the NHS are informed that their data will be used for analyses for patient benefit, to improve clinical practice and patient safety.

### BP Measurements

The GBPC used specialist hypertension nurses who are experienced and highly trained in BP measurement and follow contemporary guidelines (https://bihsoc.org/resources/bp-measurement/measure-blood-pressure/). The procedure required subjects to rest for 5 minutes in a seated position before BP was manually measured using standard sphygmomanometers. BP devices validated by the British and Irish Hypertension Society were used for BP measurements at the clinic. Clinical practice was refreshed with publication of new guidelines from the British and Irish Hypertension Society during the duration of the study. Three BP measurements were performed, 1 minute apart, and the mean of the second and third measurements was recorded. Patients attending the clinic were advised to take their regular medications as usual. Each patient attended the same clinic and would have their BP measured in the same 3-hour window either in the morning or evening on each visit. Drug substitution, addition, and dose adjustment occurred during follow-up and was in accordance with clinical guidelines. Prescribed medications were cross-checked with patients at each clinic visit, and adherence with treatment was strongly advised. However, dietary intake and the level of physical exertion before each clinic appointment could not be controlled. A more accurate measure of the time between drug consumption and BP measurements was unavailable. Information on specific medications, their doses, and formal concordance testing was unavailable.

In addition to baseline BP, we calculated average BP over 2 longitudinal time periods: year 1 and years 2 to 5. To be included in each time frame, subjects were required to have a minimum of 3 BP readings ≥30 days apart. We calculated the mean BP for each time frame using a time-weighted average of all BP measurements during the interval to remove any upward bias of the mean BP caused by more frequent appointments when BP was uncontrolled. Patients >25 years of age were included in the outcome analyses, which were performed in the overall group and in 2 age categories (age <60 and ≥60 years) recognizing the natural decline in DBP and a consequent increase in pulse pressure after the age of 55 years.

### Outcome and Comorbidity Assessment

Hospital admissions and mortality data were available from 1980 to March 2013 and were obtained from the NHS Information and Statistics Division. In Scotland, the NHS provides primary and secondary health care to all citizens, free at point of access. The diagnoses from the patients’ admissions were available from Information and Statistics Division according to the World Health Organization Classification of Diseases (*International Classification of Diseases–Ninth Revision* before 1996 and *International Classification of Diseases–Tenth Revision* after 1996). Comorbidities at baseline, 1-year, and 5-year follow-up were determined using the Charlson comorbidity score.^23^ The morbidity outcomes were the first admission with any cardiovascular event, myocardial infarction (MI), ischemic heart disease (IHD), cerebrovascular accident (CVA), heart failure (HF), and peripheral vascular disease. The mortality outcomes were all-cause deaths, cardiovascular deaths, IHD deaths, CVA deaths, and noncardiovascular deaths. The patients were followed from first BP clinic visit until death, emigration, or the end of follow-up on April 1, 2011.

### Statistical Analysis

Baseline and longitudinal characteristics were summarized overall and by age groups (age <60 and ≥60 years). Continuous variables were presented as mean±SD and categorical data as numbers and percentages. Comparisons between the age categories were made using either independent *t* tests for continuous variables or χ^2^ test for categorical variables.

The relationship between cause-specific morbidity and mortality events and 5-year longitudinal BP was assessed by a time-dependent, Cox proportional hazard (PH) model, in which baseline, 1-year average BP, and 2- to 5-year average BP measurements were included in the model as the major time-dependent predictor variables (ie, taking account of varying BP between subsequent visits) in univariate analysis. Data were censored when patients had a primary outcome event, died, or left Scotland. A Multivariable Cox PH model was adjusted for baseline variables, age, sex, body mass index, cholesterol, smoking status, and Charlson comorbidity index as time-dependent variable. A variable on year of the first visit strata (epochs) was used to adjust the secular trend in mortality and was divided into 5 categories (first visit, 1977 or before, between years 1978 and 1985, 1986 and 1993, 1994 and 2001, 2002, and thereafter). Time-dependent analyses were performed using time intervals at 1 and 5 years of follow-up. In addition, time-dependent postbaseline DBP was divided into 4 groups, <80, 80 to 89.9 (referrant), 90 to 99.9, and ≥100 mm Hg, and analyzed accordingly in the Cox PH model. All the above analyses were performed separately for the 2 age group categories. The multivariable adjusted relationship between BP and cause-specific events was also assessed using a time-dependent, Cox PH model with restricted cubic splines, which allows for nonlinearity without assuming any specific contour. A *P* value of <0.05 (2 sided) was considered statistically significant for all tests. Log-minus-log plots were analyzed for any suggestion of deviation from the PH assumption. We performed 2 multivariable adjusted Cox analysis: model 1 (included SBP and DBP), adjusted for age, sex, body mass index, smoking, cholesterol, SBP, and DBP as time-dependent variables, epochs and Charlson score; model 2 (excluded SBP), adjusted for age, sex, body mass index, smoking, cholesterol, and DBP as time-dependent variable, epochs and Charlson score. We also constructed cubic splines for SBP and DBP from model 1 and DBP from model 2.

Competing risk regression based on the Fine-Gray proportional subhazards model was performed, and subdistribution hazard ratio (HR) and 95% CI were calculated to estimate the risk of DBP <80 mm Hg according to cardiovascular or noncardiovascular outcomes. The cumulative incidence function was used to graph the rates of outcomes, thus accounting for the competing risk. All analyses were performed using R, version 3.5.1 (https://www.R-project.org/).

## Results

### Baseline Demographics

There were 10 355 eligible patients categorized into 2 age groups (age <60 and ≥60 years). Table 1 shows the baseline demographics and longitudinal BP characteristics of the study population. The average age was 52 years with nearly equal proportion of men and women overall. Around 15% of the overall cohort had DBP <80 mm Hg, and 29% had DBP ≥100 mm Hg. Patients with age ≥60 years had a greater proportion of women, higher SBP, lower DBP, and higher Charlson comorbidity score. Table 2 summarizes the patient characteristics by DBP categories and shows the DBP <80 mm Hg group is older with a greater proportion of women, lower proportion of smokers, and lower comorbidity scores.

**Table 1.**
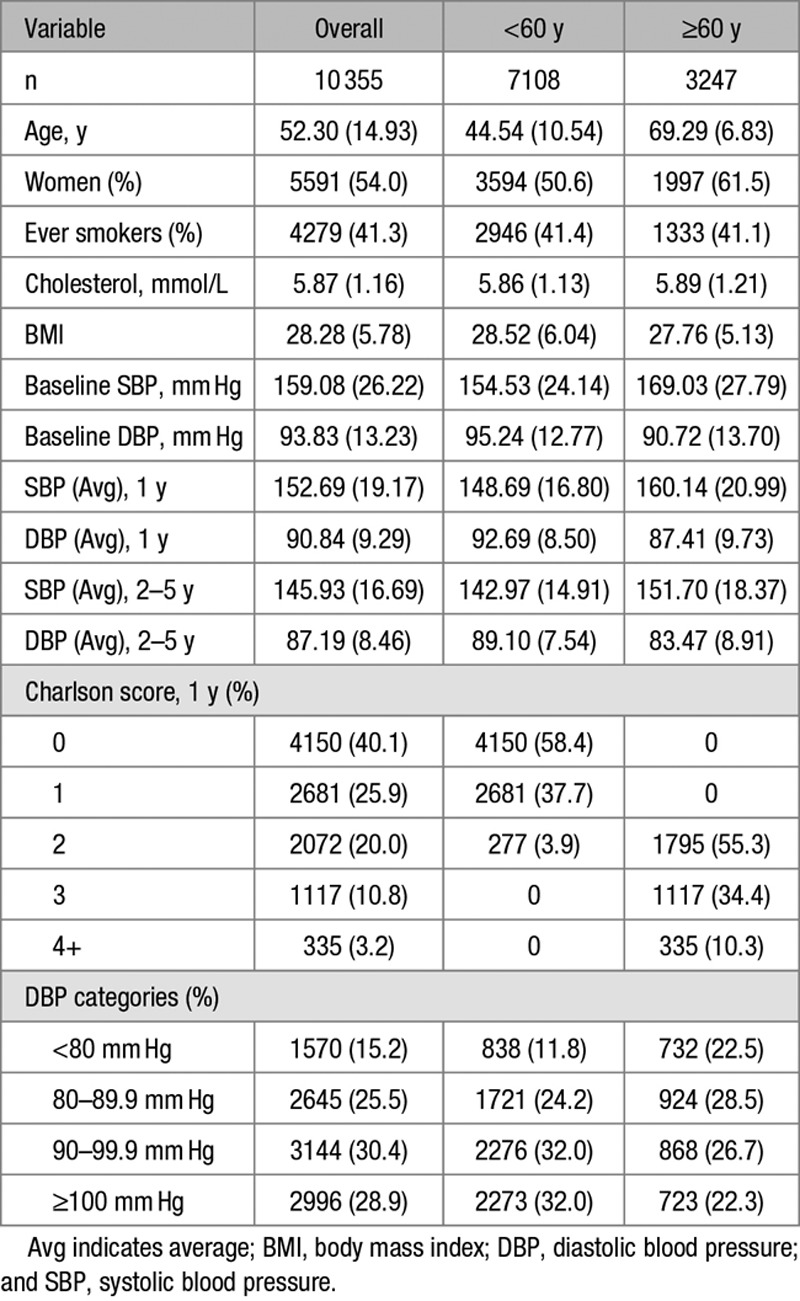
Demographics and Blood Pressure Characteristics of the Study Population

**Table 2.**
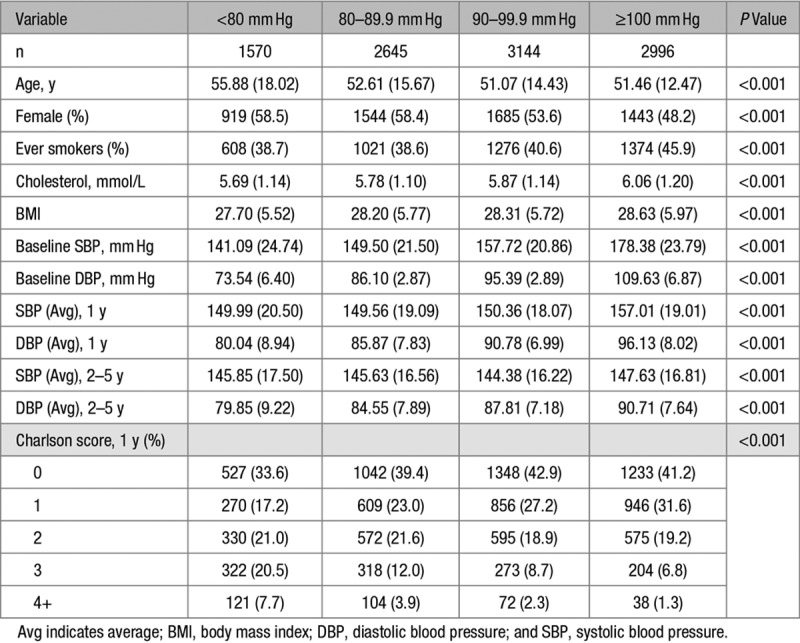
Demographics by DBP Categories at Baseline

### Outcome Analyses

In the entire cohort of 10 355 patients, there were 2956 primary outcome events (cardiovascular admissions+cardiovascular deaths) over 110 979 person-years of follow-up, 2960 all-cause deaths over 131 064 person-years, and 1299 cardiovascular deaths over 145 548 person-years. In the age <60 years subgroup, there were 1769 primary outcomes over 85 182 person-years, whereas age ≥60 years subgroup had 1187 primary outcomes over 25 796 person-years of follow-up. Table S1 in the online-only Data Supplement summarizes all-cause–specific events and person-years of follow-up.

**Figure 1.**
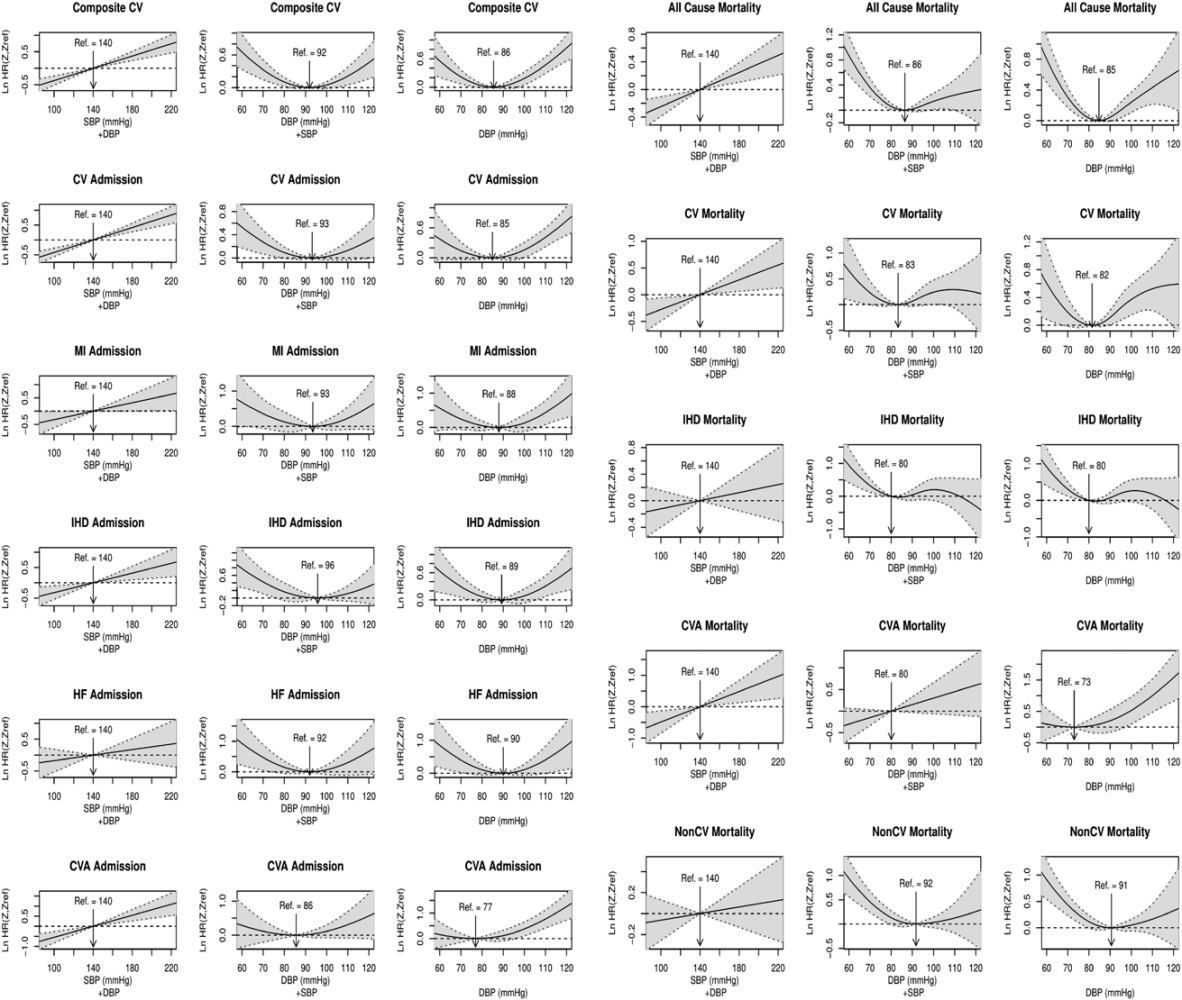
Cubic splines for the adjusted hazard ratios for time to 30-y cause-specific hospital admissions and cause-specific deaths. Shaded areas indicate 95% CIs. Three results are presented for each outcome: the relationship between systolic blood pressure (SBP) on cause-specific outcomes using model 1 where both SBP and diastolic blood pressure (DBP) are included in the model; the relationship between DBP and cause-specific outcomes using model 1 where both SBP and DBP are included in the model; and the relationship between DBP and cause-specific outcomes using model 2 where SBP is excluded from the model. CV indicates cardiovascular; CVA, cerebrovascular accident; HF, heart failure; IHD, ischemic heart disease; LnHR, logarithm of hazard ratio; and MI, myocardial infarction.

**Figure 2.**
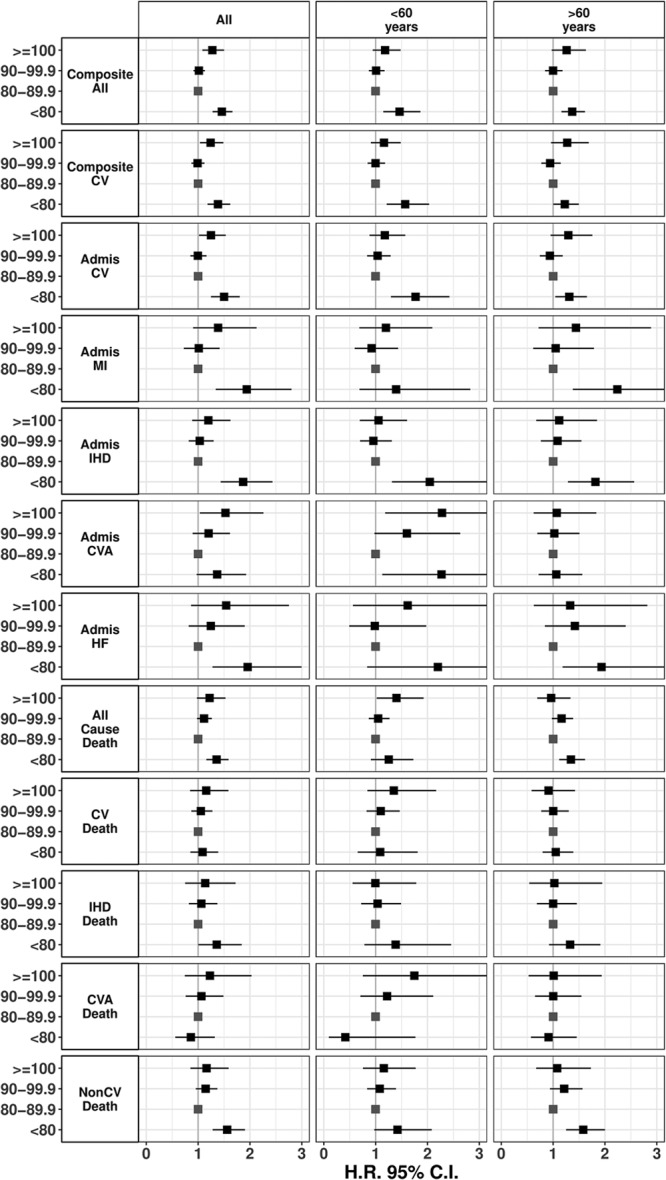
Forest plot of hazard ratios (HRs) with 95% CI for 30-y cause events by diastolic blood pressure categories (model 1). All HRs were calculated using diastolic blood pressure of 80 to 89.9 mm Hg as the referrant category. The left column presents the results from the overall cohort analysis while the middle and right columns show the results for the subgroups <60 y and >60 y, respectively. CV indicates cardiovascular; CVA, cerebrovascular accident; HF, heart failure; IHD, ischemic heart disease; and MI, myocardial infarction.

We performed 2 multivariable adjusted Cox analyses: one with adjustment for SBP as time-dependent variable (model 1) and the second excluding SBP (model 2), along with other covariates. We constructed cubic splines for SBP and DBP from model 1 and DBP from model 2, and the plots are presented in Figure 1. There was a clear linear relationship between higher SBP and greater risk of the primary cardiovascular outcome, all nonfatal cardiovascular events except HF admissions, all-cause mortality, and cardiovascular mortality. In model 2 where the effect of SBP is not accounted for, there is a substantial increase in risk at higher levels of DBP for all outcomes except noncardiovascular and IHD mortality. When SBP is accounted for in the model, the effects are specific for DBP, and these results are described in more detail below and full results presented in Figures 1 and 2 and Table S2.

We describe the observed nonlinear relationships between DBP and outcomes using 3 terms—U shaped, if the relationship is symmetrical across a nadir DBP level with increasing risks at higher and lower levels of DBP from the nadir; J shaped, if the relationship is asymmetrical with clear augmentation of risk with increasing DBP from the nadir; reverse J shaped, for an asymmetrical nonlinear relationship with clear augmentation of risk with decreasing DBP from the nadir. For the primary composite cardiovascular outcome (cardiovascular admissions+cardiovascular deaths), the relationship with DBP was U shaped with the nadir of risk at 92 (model 1) or 86 mm Hg (model 2) and substantially and significantly increased HRs for the highest and lowest DBP groups (Figure 1). The HRs for the primary cardiovascular outcome after adjustment for SBP (model 1) were 1.38 (95% CI, 1.18–1.62) and 1.24 (95% CI, 1.04–1.49) for DBP <80 and DBP ≥100, respectively, compared with reference DBP 80 to 90 group (Figure 1; Table S2). In contrast, subgroup age <60 and ≥60 years showed, respectively, a strong reverse J-shaped and a weak U-shaped relationship for the primary outcome. For subgroup age <60 years, the HRs for DBP <80 and DBP ≥100 were 1.57 (95% CI, 1.21–2.03) and 1.16 (95% CI, 0.91–1.49), respectively, whereas for age ≥60 years, they were 1.23 (95% CI, 1–1.5) and 1.27 (95% CI, 0.96–1.69), respectively.

For cardiovascular admissions, the relationship with DBP was U-shaped (nadir at DBP of 93 mm Hg) with model 1 but J-shaped relationship with model 2 (nadir at DBP of 85 mm Hg; Figure 1). The U-shaped relationship evident for cardiovascular admissions reflects the net contribution different risk profiles of its constituents—reverse J shaped for MI, IHD, and HF admissions but J/U shaped for CVA admissions (Figure 1; Table S2). Stratifying by age, both age <60 and ≥60 years show J-shaped relationships with different patterns of contributions from their constituent outcomes. For the age <60 subgroup, MI and HF admissions showed no relationship with DBP while IHD admissions showed a clear reverse J-shaped relationship while CVA admissions showed a clear U-shaped relationship. For the age ≥60 subgroup, a clear reverse J-shaped relationship was seen for MI, IHD, and HF admissions while CVA admissions showed no association with DBP categories.

Mortality analyses showed a reverse J-shaped relationship for all-cause mortality with DBP, primarily because DBP< 80 group associated with substantially and significantly increased risk of noncardiovascular mortality (1.56 [95% CI, 1.28–1.9]) and a slightly higher risk for IHD mortality (1.36 [95% CI, 1.01–1.84]) compared with the referrant group (Figure 2; Table S2). The mortality results were primarily driven by age ≥60 subgroup, which showed strong reverse J-shaped relationship for all-cause mortality and noncardiovascular mortality with comparable HRs to the overall analysis. Age <60 subgroup showed a significantly increased risk only for all-cause mortality in the DBP ≥100 category (1.4 [95% CI, 1.02–1.93]).

### Competing Risk Analysis

**Figure 3.**
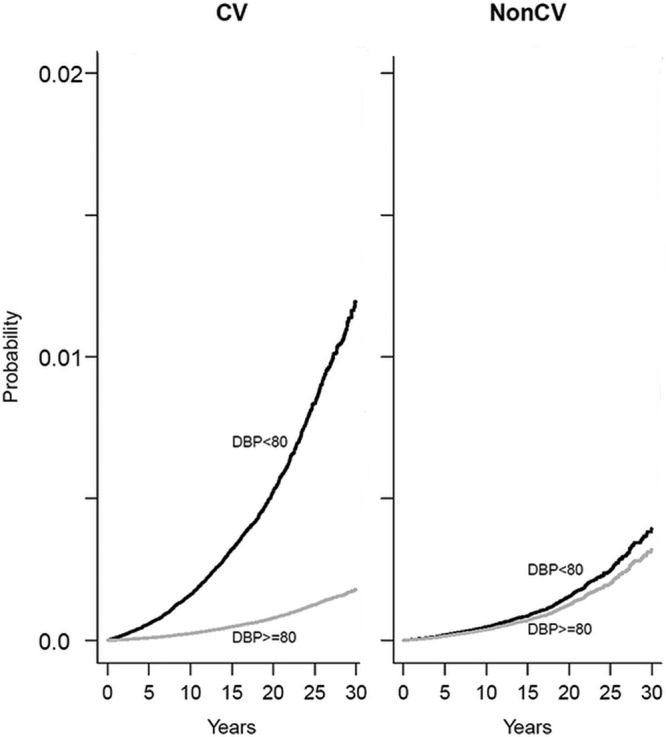
Cumulative incidence functions for primary cardiovascular (CV) outcome and non-CV mortality by diastolic blood pressure (DBP) categories.

Figure 3 shows the estimated cumulative incidence functions for DBP <80 and DBP ≥80 mm Hg based on model 1 for composite cardiovascular outcomes and noncardiovascular mortality indicating the increased risk associated with DBP <80 mm Hg for both cardiovascular and noncardiovascular outcomes. DBP <80 mm Hg was associated with increased risk of cardiovascular outcomes (subdistribution hazard, subdistribution HR, 1.33; 95% CI, 1.169–1.51; *P*=1.46×10^−5^), and these estimates were comparable to the cause-specific hazards obtained from the traditional Cox PH analyses.

## Discussion

The results of this observational study of 10 355 treated hypertensive patients extends and adds to current understanding of the diastolic J curve in hypertension. As expected, we observed evidence for a linear relationship for SBP and a nonlinear relationship for DBP with a range of cardiovascular outcomes. Our results show that while DBP <80 mm Hg is associated with increased risk for first admissions with IHD, MI, and HF, it does not translate to increased cardiovascular mortality. After stratifying by age, DBP <80 mm Hg is associated with increased risk of admissions with IHD and HF in both the older and younger age groups; however increased risk of CVA was evident only in the younger subgroup (<60 years). We observed an increased risk of noncardiovascular mortality with DBP <80 mm Hg, which was surprising. Competing risk analysis confirmed increased risk associated with DBP <80 mmHg for cardiovascular outcomes after accounting for the risk of noncardiovascular mortality. Our results suggest that the short-term adverse cardiovascular impact of intensive DBP lowering does not translate into long-term mortality risk, and one possible reason may be the long-term beneficial effect of the concomitant low SBP that accompanies low DBP. The surprising result of increased noncardiovascular mortality with low DBP may indicate reverse causality, and this warrants further study.

The J-curve controversy stems from observations of a nonlinear relationship between DBP and cardiovascular risk.^24^ Additionally, there is a specific diastolic J-curve relationship, which is evident in the presence of coronary artery disease and explained by limited coronary flow reserve.^4^ While there is consistent evidence from a majority of studies in hypertensive patients that lowering DBP (as defined by achieved BP or on-treatment BP <70) is associated with increased risk of coronary events,^14,16–19,25^ this relationship was not evident in a study of 1.2 million patients not selected for hypertension.^15^ For mortality outcomes, the results of our study concur with SYS-EUR (Systolic Hpertension in Europe Trial),^25^ which also studied treated hypertensive patients, but not with the CLARIFY registry, which analyzed patients with CAD and hypertension.^17^ The Framingham study of recurrent CVD in subjects with isolated systolic hypertension and prevalent CVD showed an association of recurrent CVD with DBP <70 irrespective of pulse pressure levels.^16^ The key criticism against the J-curve phenomenon is reverse causality, where the higher risk seen with low DBP may be due to severe underlying chronic conditions including cancer or HF, which are associated with low BPs. The HOT study (Hypertension Optimal Treatment^26^; age, 50–80 years) indicated that the J-curve relationship between DBP and coronary events could be explained by underlying pathology such as poor LV function, poor general health, and artery stiffness. A post hoc analysis of SPRINT (Systolic BP Intervention Trial),^27^ which included only hypertensive subjects over the age of 50 years, showed a U-shaped relationship between baseline DBP and the primary composite outcome, but intensive SBP lowering showed reduced risk across all baseline quintiles of DBP. Our results concur with the results of SPRINT for composite cardiovascular outcomes among the age ≥60 years group. Additionally, our study demonstrates the higher risk for nonfatal cardiovascular events with low DBP is prominent in the younger age group, which is not represented in the SPRINT study.

Additionally, higher pulse pressure reflecting increased arterial stiffness may partially explain the J-curve phenomenon for DBP. While general population cohorts (Framingham^14^: age, 30–62 years; CALIBER^15^: age, 30–80 years) showed a J-shaped relationship only in those with high pulse pressure, a reanalysis of the INVEST trial (International Verapamil SR-Trandolapril)^28^ (age, 66±10 years), REACH registry (Reduction of Atherothrombosis for Continued Health)^29^ (age, 68±10 years), and CLARIFY^17^ (age, 65±10 years) showed increased cardiovascular risk for both high and low pulse pressure. Recent intensive BP-lowering trials such as ACCORD (Action to Control Cardiovascular Risk in Diabetes)^8^ and SPRINT^7^ did not show a J curve between BP and outcomes. A reanalysis of the TNT trial (Treating to New Targets) showed that in patients with coronary artery disease, a J- or U-shaped relationship with SBP and DBP was present.^6^ In this study, all patients who had coronary artery disease were 61 years of age and were not on antihypertensives. It is well known that DBP decreases after the age of 55 years with consequent increase in pulse pressure in older patients, and hence age may explain some of the variability in results. Because age is the strongest marker of pulse pressure, we tried to overcome this by analyzing patients <60 and >60 years of age separately in our study. Our finding of the J curve in both older and younger subgroups for IHD admissions supports the adverse impact of intensive DBP lowering on cardiovascular risk but not mortality.

Comparable with previous studies, we did not observe a nonlinear relationship for CVA mortality in either age group and for cardiovascular admissions in age ≥60 group. However, age <60-year subgroup showed a clear U-shaped relationship between DBP and CVA admissions. This novel finding suggests analyses without stratifying by age or studies of cohorts >60 years may attribute a neutral risk from DBP on CVA risk. CVA death showed a linear relationship with DBP in the age <60-year subgroup and neutral effects in the older subgroup.

The ARIC study (Atherosclerosis Risk in Communities)^30^ showed those with DBP of 60 to 69 mm Hg had a higher prevalence of baseline high-sensitivity cardiac troponin >14 ng/L compared with those with DBP of 80 to 89.9 mm Hg. This suggests that low DBP might be causing ongoing subclinical myocardial damage. Our results in this context may reflect unmasking of ongoing myocardial damage by pharmacological lowering of DBP to <80 mm Hg by the increased incidence of IHD and HF admissions. However, further research is required to establish the best screening method, and studies of high-sensitivity cardiac troponin I and NT-proBNP (N-terminal pro-B-type natriuretic peptide) as potential safety biomarkers for screening during intensive BP reduction are attractive options.^31–33^

Ours is an observational study, so we cannot fully address reverse causality. Nevertheless, to explore patterns in association that would shed insight into the J-curve phenomenon, we conducted separate analyses for patients >60 and <60 years of age and additionally adjusted for comorbidities at baseline, 1 year, and 5 years of follow-up. We show cause- and age-specific differences in the pattern of nonlinear association between DBP and cardiovascular outcomes. Low DBP is significantly associated with MI and HF admissions only in the older subgroup and CVA in the younger subgroup. While we did not see a strong association between low DBP and cardiovascular mortality, there was a borderline increased risk of IHD mortality with DBP <80. Importantly, CVA deaths showed a consistent linear trend for increased risk with increasing DBP. Our study highlights the complex nature of DBP on cardiovascular outcomes, which may partially explain variable results from previous studies and should inform future studies.

The main strengths of our study are the analysis of a large cohort of real-world treated hypertensive subjects with ≤30-year follow-up, high event rates, and analysis of cause-specific morbidity and mortality outcomes. We also addressed reverse causality and the effect of pulse pressure by stratifying our analysis by age and analyzing cause-specific morbidity and mortality separately. The main limitations are single-center secondary/tertiary-care cohort, observational nature of the study, lack of data on patient adherence, physician inertia, use of clinic BP and not out-of-office measurements. We used health record linkage across the NHS in Scotland to detect cardiovascular admission and mortality events. While this would not pick up events occurring outside Scotland, data from randomized clinical trials conducted in Scotland using record linkage, such as WOSCOPS (West of Scotland Coronary Prevention Study), show a low level of underreporting in record linkage data and suggest that this is not a major issue.^34^ Indeed, these data indicate that cardiovascular outcome detection using record linkage in a clinical trial performed in a country with a unified health system, such as Scotland, would likely lead to qualitatively similar outcomes to those obtained by the current expensive clinical trial model involving rigorous individual patient follow-up and intensive data collection.^35^ Measurement of BP by the GBPC clinic nurses may have changed over the years because of changes in the British and Irish Hypertension Society measurement guidelines. We also have not adjusted for treatment differences either in terms of drug classes prescribed or treatment titration because of lack of prescription data. Baseline alcohol intake in our cohort did not show significant association with cardiovascular outcomes and may likely reflect the effect of advice that patients receive at every clinic visit on lifestyle modification and limiting alcohol intake. The generalizability of our findings needs to be confirmed through independent studies.

## Perspectives

Increased cardiovascular morbidity attributable to lowering DBP <80 mmHg in treated hypertensive patients does not translate to increased cardiovascular mortality. Evolving hypertension treatment guidelines recommend more intensive BP reduction may lead to unintended consequences of higher healthcare utilization because of increased cardiovascular morbidity, and this merits future prospective studies. For reasons unclear, low on-treatment DBP is associated with increased risk of noncardiovascular mortality, and this merits further study.

## Sources of Funding

S. Padmanabhan is funded by the Medical Research Council (MR/M016560/1; AIM-HY Study) and the British Heart Foundation (BHF; PG/12/85/29925; CS/16/1/31878). L. McCallum is funded by BHF (FS/14/52/30901). A.F. Dominiczak has funding from the Scottish Ecosystem for Precision Medicine.

## Disclosures

None.

## Supplementary Material

## Novelty and Significance

### What Is New?

- Low diastolic blood pressure (BP) is associated with increased risk of cardiovascular morbidity but not of cardiovascular mortality in both older and younger hypertensive patients.

### What Is Relevant?

- The diastolic J curve needs to be considered in the management of treated hypertensive patients. Intensive diastolic BP lowering may not be associated with long-term mortality risks.

### Summary

There is still a need for another adequately powered randomized controlled trial of high-risk hypertensive patients randomized to target diastolic BP <90, <80, and <70 mm Hg to establish the nonlinear relationship between diastolic BP and outcomes. Evolving new hypertension guidelines promoting lower systolic BP targets will require prospective monitoring to assess the impact on morbidity and cost-effectiveness. Whether additional cardiovascular monitoring is required to safely achieve lower BP targets warrants further study.

## References

[1] Forouzanfar MH, Liu P, Roth GA (2017). Global burden of hypertension and systolic blood pressure of at Least 110 to 115 mm Hg, 1990-2015.. JAMA.

[2] Mancia G, Fagard R, Narkiewicz K (2013). 2013 ESH/ESC guidelines for the management of arterial hypertension: the Task Force for the Management of Arterial Hypertension of the European Society of Hypertension (ESH) and of the European Society of Cardiology (ESC).. Eur Heart J.

[3] Stewart IM (1979). Relation of reduction in pressure to first myocardial infarction in patients receiving treatment for severe hypertension.. Lancet.

[4] Cruickshank JM (1988). Coronary flow reserve and the J curve relation between diastolic blood pressure and myocardial infarction.. BMJ.

[5] D’Agostino RB, Belanger AJ, Kannel WB, Cruickshank JM (1991). Relation of low diastolic blood pressure to coronary heart disease death in presence of myocardial infarction: the Framingham Study.. BMJ.

[6] Bangalore S, Messerli FH, Wun CC, Zuckerman AL, DeMicco D, Kostis JB, LaRosa JC, Treating to New Targets Steering Committee and Investigators (2010). J-curve revisited: an analysis of blood pressure and cardiovascular events in the Treating to New Targets (TNT) Trial.. Eur Heart J.

[7] Wright JT, Williamson JD, Whelton PK, SPRINT Research Group (2015). A randomized trial of intensive versus standard blood-pressure control.. N Engl J Med.

[8] Cushman WC, Evans GW, Byington RP, ACCORD Study Group (2010). Effects of intensive blood-pressure control in type 2 diabetes mellitus.. N Engl J Med.

[9] Benavente OR, Coffey CS, Conwit R, Hart RG, McClure LA, Pearce LA, Pergola PE, Szychowski JM, SPS3 Study Group (2013). Blood-pressure targets in patients with recent lacunar stroke: the SPS3 randomised trial.. Lancet.

[10] MacMahon S, Peto R, Cutler J, Collins R, Sorlie P, Neaton J, Abbott R, Godwin J, Dyer A, Stamler J (1990). Blood pressure, stroke, and coronary heart disease. Part 1, Prolonged differences in blood pressure: prospective observational studies corrected for the regression dilution bias.. Lancet.

[11] Hansson L, Zanchetti A, Carruthers SG, Dahlöf B, Elmfeldt D, Julius S, Ménard J, Rahn KH, Wedel H, Westerling S (1998). Effects of intensive blood-pressure lowering and low-dose aspirin in patients with hypertension: principal results of the Hypertension Optimal Treatment (HOT) randomised trial. HOT Study Group.. Lancet.

[12] Greenberg JA (2002). Hypothesis - the J-shaped follow-up relation between mortality risk and disease risk-factor is due to statistical confounding.. Med Hypotheses.

[13] Marschner IC, Simes RJ, Keech A (2007). Biases in the identification of risk factor thresholds and J-curves.. Am J Epidemiol.

[14] Kannel WB, Wilson PW, Nam BH, D’Agostino RB, Li J (2004). A likely explanation for the J-curve of blood pressure cardiovascular risk.. Am J Cardiol.

[15] Rapsomaniki E, Timmis A, George J, Pujades-Rodriguez M, Shah AD, Denaxas S, White IR, Caulfield MJ, Deanfield JE, Smeeth L, Williams B, Hingorani A, Hemingway H (2014). Blood pressure and incidence of twelve cardiovascular diseases: lifetime risks, healthy life-years lost, and age-specific associations in 1·25 million people.. Lancet.

[16] Franklin SS, Gokhale SS, Chow VH, Larson MG, Levy D, Vasan RS, Mitchell GF, Wong ND (2015). Does low diastolic blood pressure contribute to the risk of recurrent hypertensive cardiovascular disease events? The Framingham Heart Study.. Hypertension.

[17] Vidal-Petiot E, Greenlaw N, Ford I, Ferrari R, Fox KM, Tardif JC, Tendera M, Parkhomenko A, Bhatt DL, Steg PG (2018). Relationships between components of blood pressure and cardiovascular events in patients with stable coronary artery disease and hypertension.. Hypertension.

[18] Böhm M, Schumacher H, Teo KK, Lonn EM, Mahfoud F, Mann JFE, Mancia G, Redon J, Schmieder RE, Sliwa K, Weber MA, Williams B, Yusuf S (2017). Achieved blood pressure and cardiovascular outcomes in high-risk patients: results from ONTARGET and TRANSCEND trials.. Lancet.

[19] Böhm M, Schumacher H, Teo KK, Lonn E, Mahfoud F, Mann JFE, Mancia G, Redon J, Schmieder R, Weber M, Sliwa K, Williams B, Yusuf S (2018). Achieved diastolic blood pressure and pulse pressure at target systolic blood pressure (120-140 mm Hg) and cardiovascular outcomes in high-risk patients: results from ONTARGET and TRANSCEND trials.. Eur Heart J.

[20] Whelton PK, Carey RM, Aronow WS (2018). 2017 ACC/AHA/AAPA/ABC/ACPM/AGS/APhA/ASH/ASPC/NMA/PCNA guideline for the prevention, detection, evaluation, and management of high blood pressure in adults: A Report of the American College of Cardiology/American Heart Association Task Force on Clinical Practice Guidelines.. Hypertension.

[21] Williams B, Mancia G, Spiering W, ESC Scientific Document Group (2018). 2018 ESC/ESH guidelines for the management of arterial hypertension.. Eur Heart J.

[22] Lip S, Jeemon P, McCallum L, Dominiczak AF, McInnes GT, Padmanabhan S (2016). Contrasting mortality risks among subgroups of treated hypertensive patients developing new-onset diabetes.. Eur Heart J.

[23] Quan H, Sundararajan V, Halfon P, Fong A, Burnand B, Luthi JC, Saunders LD, Beck CA, Feasby TE, Ghali WA (2005). Coding algorithms for defining comorbidities in ICD-9-CM and ICD-10 administrative data.. Med Care.

[24] Kjeldsen SE, Oparil S, Narkiewicz K, Hedner T (2016). The J-curve phenomenon revisited again: SPRINT outcomes favor target systolic blood pressure below 120 mm Hg.. Blood Press.

[25] Fagard RH, Staessen JA, Thijs L, Celis H, Bulpitt CJ, de Leeuw PW, Leonetti G, Tuomilehto J, Yodfat Y (2007). On-treatment diastolic blood pressure and prognosis in systolic hypertension.. Arch Intern Med.

[26] Hansson L (2000). Antihypertensive treatment: does the J-curve exist?. Cardiovasc Drugs Ther.

[27] Beddhu S, Chertow GM, Cheung AK, SPRINT Research Group (2018). Influence of baseline diastolic blood pressure on effects of intensive compared with standard blood pressure control.. Circulation.

[28] Bangalore S, Messerli FH, Franklin SS, Mancia G, Champion A, Pepine CJ (2009). Pulse pressure and risk of cardiovascular outcomes in patients with hypertension and coronary artery disease: an International Verapamil SR-Trandolapril Study (INVEST) analysis.. Eur Heart J.

[29] Selvaraj S, Steg PG, Elbez Y, Sorbets E, Feldman LJ, Eagle KA, Ohman EM, Blacher J, Bhatt DL, REACH Registry Investigators (2016). Pulse pressure and risk for cardiovascular events in patients with atherothrombosis: from the REACH Registry.. J Am Coll Cardiol.

[30] McEvoy JW, Chen Y, Rawlings A, Hoogeveen RC, Ballantyne CM, Blumenthal RS, Coresh J, Selvin E (2016). Diastolic blood pressure, subclinical myocardial damage, and cardiac events: implications for blood pressure control.. J Am Coll Cardiol.

[31] Welsh P, Poulter NR, Chang CL, Sever PS, Sattar N, ASCOT Investigators (2014). The value of N-terminal pro-B-type natriuretic peptide in determining antihypertensive benefit: observations from the Anglo-Scandinavian Cardiac Outcomes Trial (ASCOT).. Hypertension.

[32] Pokharel Y, Sun W, de Lemos JA (2015). High-sensitivity troponin T and cardiovascular events in systolic blood pressure categories: atherosclerosis risk in communities study.. Hypertension.

[33] Pokharel Y, Sun W, Villareal DT, Selvin E, Virani SS, Ndumele CE, Hoogeveen RC, Coresh J, Boerwinkle E, Butler KR, Solomon SD, Pankow JS, Bozkurt B, Ballantyne CM, Nambi V (2017). Association between high-sensitivity troponin T and cardiovascular risk in individuals with and without metabolic syndrome: The ARIC study.. Eur J Prev Cardiol.

[34] Ford I, Murray H, McCowan C, Packard CJ (2016). Long-term safety and efficacy of lowering low-density lipoprotein cholesterol with statin therapy: 20-year follow-up of West of Scotland Coronary Prevention Study.. Circulation.

[35] Barry SJ, Dinnett E, Kean S, Gaw A, Ford I (2013). Are routinely collected NHS administrative records suitable for endpoint identification in clinical trials? Evidence from the West of Scotland Coronary Prevention Study.. PLoS One.

