# Supplementary figures and images for: Diastolic Blood Pressure J-Curve Phenomenon in a Tertiary-Care Hypertension Clinic
Source: Hypertension. 2019 Aug 19;74(4):767–75. doi: 10.1161/HYPERTENSIONAHA.119.12787 (PMC6756261; doi:10.1161/HYPERTENSIONAHA.119.12787)

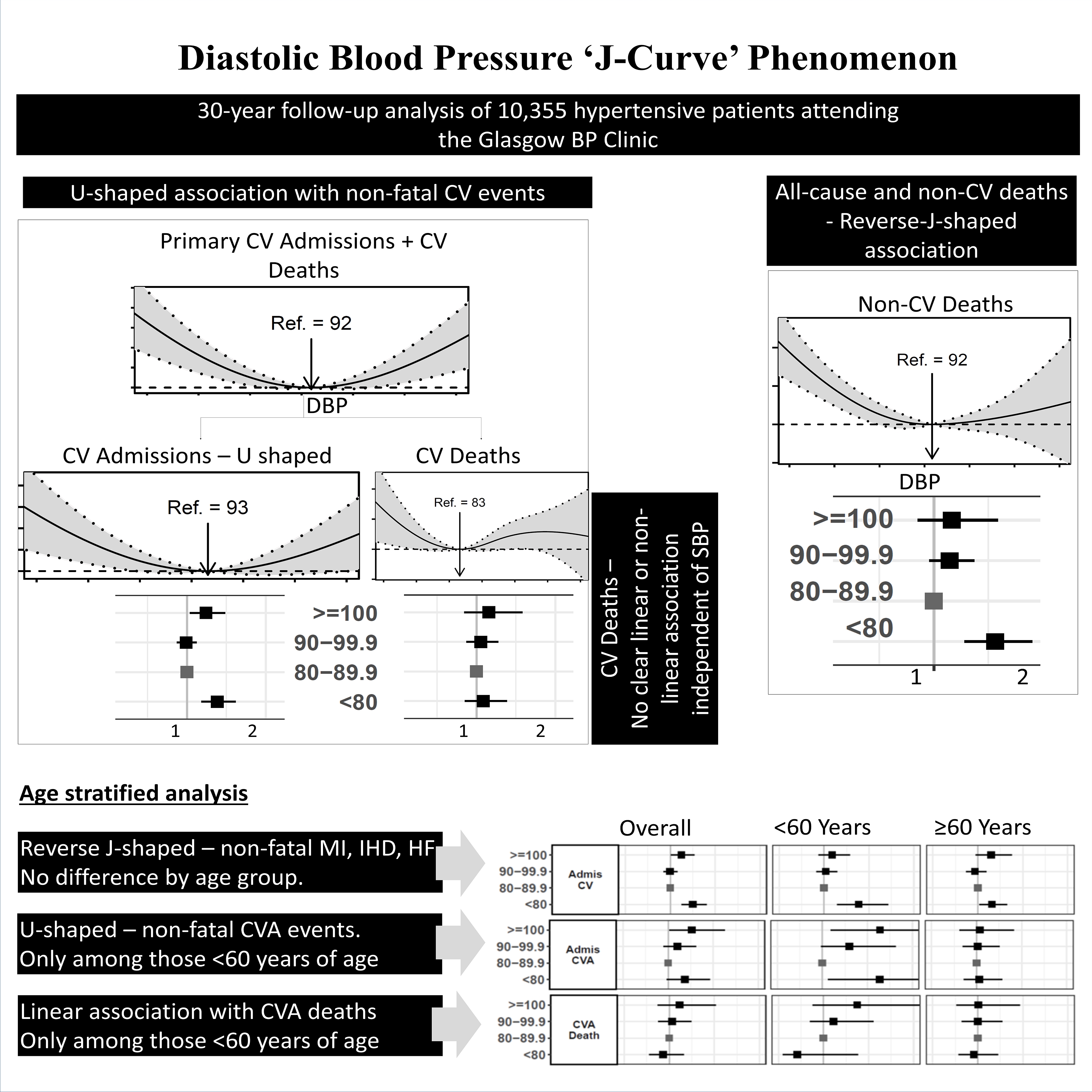

Supplement: Supplementary file 1 [file hyp-74-0767-s001.jpg]
